# Epigenetic Changes in Host Ribosomal DNA Promoter Induced by an Asymptomatic Plant Virus Infection

**DOI:** 10.3390/biology9050091

**Published:** 2020-04-28

**Authors:** Miryam Pérez-Cañamás, Elizabeth Hevia, Carmen Hernández

**Affiliations:** Instituto de Biología Molecular y Celular de Plantas (Consejo Superior de Investigaciones Científicas-Universidad Politécnica de Valencia), Ciudad Politécnica de la Innovación, Ed. 8E. Camino de Vera s/n, 46022 Valencia, Spain

**Keywords:** RNA virus, DNA methylation, transcriptional gene silencing, plant virus, METHYLTRASFERASE 1, demethylases, viral suppressor of RNA silencing, *Tombusviridae*

## Abstract

DNA cytosine methylation is one of the main epigenetic mechanisms in higher eukaryotes and is considered to play a key role in transcriptional gene silencing. In plants, cytosine methylation can occur in all sequence contexts (CG, CHG, and CHH), and its levels are controlled by multiple pathways, including de novo methylation, maintenance methylation, and demethylation. Modulation of DNA methylation represents a potentially robust mechanism to adjust gene expression following exposure to different stresses. However, the potential involvement of epigenetics in plant-virus interactions has been scarcely explored, especially with regard to RNA viruses. Here, we studied the impact of a symptomless viral infection on the epigenetic status of the host genome. We focused our attention on the interaction between *Nicotiana benthamiana* and *Pelargonium line pattern virus* (PLPV, family *Tombusviridae*), and analyzed cytosine methylation in the repetitive genomic element corresponding to ribosomal DNA (rDNA). Through a combination of bisulfite sequencing and RT-qPCR, we obtained data showing that PLPV infection gives rise to a reduction in methylation at CG sites of the rDNA promoter. Such a reduction correlated with an increase and decrease, respectively, in the expression levels of some key demethylases and of MET1, the DNA methyltransferase responsible for the maintenance of CG methylation. Hypomethylation of rDNA promoter was associated with a five-fold augmentation of rRNA precursor levels. The PLPV protein p37, reported as a suppressor of post-transcriptional gene silencing, did not lead to the same effects when expressed alone and, thus, it is unlikely to act as suppressor of transcriptional gene silencing. Collectively, the results suggest that PLPV infection as a whole is able to modulate host transcriptional activity through changes in the cytosine methylation pattern arising from misregulation of methyltransferases/demethylases balance.

## 1. Introduction

Viruses, as obligate intracellular parasites, establish complex relationships with their hosts [1,2]. This type of relationship imposes numerous challenges on both the infecting virus and the host partner [3,4]. On the virus side, it frequently usurps or alters distinct signaling pathways and cellular processes in order to sustain its multiplication, survival, and spread [5,6]. The host, on its side, deploys a set of defense mechanisms against the virus in an attempt to restrict or block the infection process [7]. In such a scenario, it is not surprising that hosts suffering from active virus infections exhibit important physiological, biochemical, and metabolic changes that in some occasions, though not always, lead to symptom development [8]. These changes may imply alterations in host gene expression at both transcriptional and post-transcriptional levels [9,10].

Epigenetic changes, such as DNA methylation and histone modification, play a central role in transcriptional reprogramming [11,12,13]. Most of the DNA methylation observed in eukaryotes is linked to cytosines, particularly those followed by guanine (CG), though in plants cytosine methylation can occur outside the CG context, including CHG and CHH (where H represents A, C, or T) [14,15]. The pattern of DNA methylation is the result of the balance between methylation and demethylation activities. In plants, de novo establishment and strengthening of DNA methylation is mediated by a plant-specific pathway known as RNA-directed DNA methylation (RdDM) [16]. In this pathway, RNA polymerase IV (Pol IV) produces transcripts that are copied by RNA-dependent RNA polymerase 2 (RDR2) into double-stranded (ds) RNAs, which, in turn, are processed by Dicer-like 3 (DCL3) into 24-nt small interfering RNAs (siRNAs). One strand of these siRNAs is loaded onto an Argonaute (AGO) protein (mainly AGO4) to form an RNA-induced silencing complex, which binds, through sequence complementarity, to nascent long noncoding transcripts generated by Pol V. The Pol V complex subsequently recruits other downstream components of RdDM, chief among which is DOMAINS REARRANGED METHYLTRANSFERASE (DRM2), to the corresponding nuclear target locus, resulting in de novo DNA methylation of such a locus in any context (CG, CHG, or CHH) [17]. Methylation at symmetric CG and CHG contexts can be maintained during DNA replication by DNA METHYLTRANSFERASE 1 (MET1) and CHROMOMETHYLASE 3 (CMT3), respectively, whereas the maintenance of methylation at the asymmetric CHH context requires persistent de novo methylation during every cell cycle. On the other side, plants possess an active DNA demethylation system catalyzed by the DNA glycosylase family of DNA demethylases [18,19,20]. Three DNA demethylases are believed to account for all demethylase activity in the somatic tissues of *Arabidopsis*, REPRESSOR OF SILENCING 1 (ROS1, also known as Demeter-like 1 or DML1), which is the most highly expressed, DML2, and DML3.

Alterations in host gene expression at the transcriptional level linked to changes in epigenetic marks have been reported in distinct plant–microbe interactions [21,22,23]. However, the potential role of epigenetics in plant–virus interactions is only starting to be glimpsed. Several studies have provided data on plant epigenome variations in response to geminiviruses, viral agents whose DNA genomes are challenged by the host DNA methylation-mediated gene silencing defense and must develop strategies to counter it [24,25,26]. In contrast, few studies have looked at the effect of RNA virus infection on host DNA methylation. Some reports have shown that RNA virus-based vectors carrying inserts corresponding to segments of gene promoters (either from transgenes or endogenous genes) are able to induce cytosine methylation of cognate promoters [21,27,28,29]. However, potential epigenetic changes induced by genuine infections of unmodified plant RNA viruses have scarcely been investigated [30,31,32]. Here, we aimed to improve our understanding of this underexplored issue using the plant–virus combination composed by *Nicotiana benthamiana*–*Pelargonium line pattern virus* (PLPV). PLPV is a member of the genus *Pelarspovirus* within the broad family Tombusviridae [33]. Its monopartite genome contains five open reading frames encoding two proteins involved in viral replication (p27 and p87), two proteins involved in viral movement, and a protein (p37) with a dual role as a coat protein and RNA silencing suppressor (VSR) [34,35,36]. PLPV infections are restricted to *Pelargonium* spp. in nature, but the virus can be transmitted experimentally to numerous plant species, including *N. benthamiana*. In the latter one as well as in its natural and in other experimental hosts, PLPV infections are usually symptomless and are characterized by low viral titers [37,38,39]. In this work, we wondered whether PLPV infection could give rise to significant alterations in the epigenetic marks of the host genome. We focused our attention on the methylation state of a selected genomic region, more particularly that corresponding to the 45S ribosomal (r) DNA whose transcriptional activity is known to be regulated by different mechanisms, including epigenetic modifications [40]. We also analyzed the expression levels of some host components involved in cytosine methylation/demethylation in order to search for potential correlations between such expression levels and the ratio of methylated cytosines. In addition, we checked whether changes in the methylation state of rDNA correlate with rRNA precursor levels. Finally, we explored whether the activity of PLPV VSR per se may affect rDNA methylation. The results support that an asymptomatic viral infection, such as that established by PLPV, may incite significant alterations in the transcriptional activity of rDNA units that are likely driven by modifications in the cytosine methylation pattern and that are apparently not linked to the VSR function. Potential implications of these observations for the progress of viral infection are discussed.

## 2. Materials and Methods

### 2.1. Plant Material

*N. benthamiana* plants that had reached the three-to-four-leaf stage were mechanically inoculated with full-length PLPV transcripts synthesized in vitro with T7 RNA polymerase from a pUC18-based construct [41]. Mock-inoculated *N. benthamiana* plants were used as a negative control.

For transient expression of PLPV VSR, *N. benthamiana* leaves of 6-weak-old plants (five-to-six-leaf stage) were infiltrated with *Agrobacterium tumefaciens* C58C1 strain transformed with a pMOG800-derived construct carrying the PLPV p37 gene flanked by the *Cauliflower mosaic virus* (CaMV) 35S promoter and the terminator sequence of the *Solanum tuberosum* proteinase inhibitor II gene (PoPit) [36]. Leaves agroinfiltrated with an empty pMOG800 vector were used as control.

For each experiment, leaves from independent plants were pooled. More specifically, systemic leaves 5, 6, and 7 (numbered from inoculated leaves since they are known to be highly infected as reported in [42]) were collected from either three mock or three PLPV-infected plants at 34 days post-inoculation (d.p.i.). Leaves infiltrated with *A. tumefaciens* transformed with pMOG800 or pMOG800-p37 were collected at 14 days post-infiltration (d.p.if.) from six independent plants (one leaf per plant) and mixed. Detection of PLPV infection and p37 expression was done by tissue-printing hybridization and Western blot, respectively, as described previously [42,43]. Two replicates of each type of experiments were done. Both, inoculated and agroinfiltrated plants, were maintained in plant growth chambers with controlled environmental conditions (16 h day at 23 °C and 8 h night at 19 °C and relative humidity 65% ± 7% with short spikes up to ± 15%) until leaf samples were harvested.

### 2.2. DNA Extraction, Bisulfite Treatment, and Sequencing

Total genomic DNA was extracted from *N. benthamiana* leaves using the GeneJET plant genomic DNA purification mini kit (Thermo Fisher Scientific, Madrid, Spain). Analyzed samples corresponded, on one side, to systemic leaves from PLPV-infected and mock-inoculated *N. benthamiana* plants and, on the other, to leaves agroinfiltrated with a pMOG800-derived construct for p37 expression or with an empty pMOG800 vector. Bisulfite treatment was performed by employing the EpiJet Bisulfite conversion kit (Thermo Fisher Scientific). Modified DNA was amplified by polymerase chain reaction (PCR) with the GoTaq DNA polymerase (Promega) and primers derived from the promoter region of 45S rDNA transcriptional unit (Accession No. KP824745) designed with the aid of the MethPrimer program [44]. Amplified DNA fragments were cloned into pTZ57R/T (Thermo Fisher Scientific). Two biological replicates of each type of sample were used and a minimum of 15 recombinant clones were sequenced in each case. To perform the in silico analysis of cytosine methylation, we used CyMATE, a universal software designed for sequences derived from bisulfite-treated plant DNA [45]. CyMATE quantifies pattern-specific methylation at each symmetric or asymmetric site and, moreover, performs a quality control of the sequencing data.

### 2.3. Quantitative Reverse Transcription-PCR (RT-qPCR)

Total RNA preparations were obtained from *N. benthamiana* leaves by phenol extraction and lithium precipitation [46]. Total RNA preparations with an RIN (RNA integrity number, Agilent) greater than or equal to 7 were treated with Turbo DNase (ThermoFisher) and reverse transcribed with PrimeScript RT reagent kit (Perfect Real Time, Takara) using an oligo-dT primer (1 μg of treated total RNA per reaction). Sequences of *N. benthamiana* genes involved in DNA methylation/demethylation were retrieved from http://benthgenome.qut.edu.au/. Specific gene primers for PCR amplification were designed by employing Primer-Express 2.0 software (Life Technologies SA, Madrid, Spain) and the criteria of a melting temperature ranging from 50 to 60 °C, PCR amplicon lengths of 100 to 200 bp, length of primer sequences of 19 to 25 nucleotides, and guanine-cytosine content of 40% to 60% (Supplementary Table S1). Master mix for qPCR was prepared with 5x PyroTaq EvaGreen qPCR Mix Plus (ROX) (Cultek Molecular Bioline). The protein phosphatase 2A (PP2A) gene was used as the housekeeping gene, as its expression has been shown to be stable under different experimental conditions, including viral infection [47]. The genes of interest and the reference gene had similar amplification efficiencies. Measurement of the precursor rRNA (pre-RNA) accumulation was performed in the same conditions but replacing the oligo-dT used in RT reactions by random primers. Pre-rRNA primers were designed encompassing the 18S and the internal transcribed spacer 1 (ITS1) so that pre-rRNA was specifically amplified (Supplementary Table S1). Three technical and three biological replicates were performed for each sample. The PCR reactions were analyzed using the ABI PRISM 7700 Sequence detection system (Life Technologies SA, Madrid, Spain) and evaluation of the relative expression level of each gene was carried out with the relative expression software tool (REST) designed by Qiagen (Hilden, Germany).

### 2.4. Statistical Analyses

Numerical data were expressed as mean ± standard deviation. To determine the statistical significance between the biological samples, a Student’s homoscedastic two-tailed *t*-test was used, considering significant differences as those with *p*-value < 0.05.

## 3. Results

### 3.1. PLPV Infection Is Associated with Hypomethylation of the Promoter Region of 45S rDNA Transcriptional Unit

**Figure 1:**
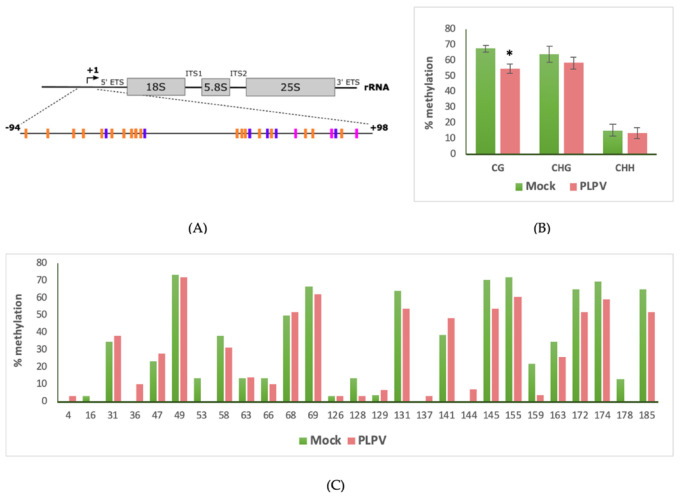
PLPV infection induces changes in the methylation pattern of rDNA promoter in *N. benthamiana*. (**A**) Diagram of the 45S rRNA transcriptional unit with the transcription start site indicated by an arrow. Stretch selected for analysis by bisulfite sequencing is detailed at the bottom, with the potential methylated cytosines labelled in pink (CG), purple (CHG), and orange (CHH). (**B**) Differential DNA methylation levels between mock-inoculated (green) and PLPV-infected plants (red) in the CG, CHG, and CHH sequence contexts. Error bars depict the standard deviations from two independent biological replicates and the statistical significance was tested using a paired *t*-test (* *p* < 0.05). (**C**) Position-specific methylation levels in the analyzed samples of rDNA.

To assess whether PLPV infection may induce alterations in the methylation state of the host genome, the 45S rDNA transcriptional unit was chosen as the target sequence. This type of unit is arranged into clusters consisting of modules of an almost identical sequence that are separated from each other by an intergenic spacer (IGS) region and encode the pre-rRNA of the three largest rRNAs, 18S, 5.8S, and 25S in plants, whose sequences are flanked by external (5’ETS and 3´ETS) and internal (ITS1 and ITS2) transcribed spacers (Figure 1). Mature rRNAs will be released from pre-rRNA after a sophisticated process leading to the removal of ETSs and ITSs [48,49].

We used a standard approach consisting in bisulfite treatment (which deaminates unmethylated cytosines and converts them into uracils) of genomic DNA samples obtained from PLPV-infected and mock control leaves. The treatment was followed by specific PCR amplification, cloning, and sequencing of PCR product(s) to analyze the presence of methylated and unmethylated cytosines in a stretch of 192 bp encompassing the promoter region of the 45S rDNA transcriptional unit (positions −94 to +98 from the +1 transcription initiation site) (Figure 1A). The examined sequence contained 8 symmetric (3 CG, 5 CHG) and 18 asymmetric (CHH) potential methylation sites. DNA samples included in the assay were obtained from systemic leaves of PLPV-infected and control mock-inoculated *N. benthamiana* plants collected at 34 d.p.i (Supplementary Figure S1). The sequences of 30 clones derived from two biological replicates were inspected for each type of sample. A PLPV-unrelated and unmethylated DNA, obtained by PCR, was used as a control in the bisulfite reactions to corroborate the completeness of the bisulfite treatment in our experimental conditions (data not shown). Methylation analysis revealed that PLPV infection resulted in a significant decrease in the relative number of methylated cytosine residues at CG context when compared with control plants (54.88% versus 67.61%) (Figure 1B,C). No significant changes in the level of cytosine methylation at the CHG or CHH contexts were detected. Thus, the results allowed us to establish an association between PLPV infection and a hypomethylated status of the analyzed rDNA promoter region at symmetric CG sites.

### 3.2. Expression Levels of Methylation/Demethylation Genes Are Misregulated in PLPV-Infected Plants

**Figure 2:**
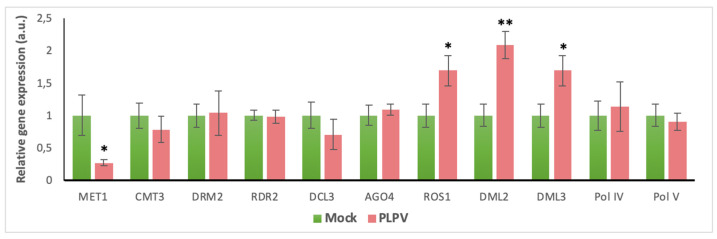
Expression levels of DNA methylation/demethylation genes during a PLPV infection. RT-qPCR analysis of mRNA levels of the selected genes in systemic leaves from mock-inoculated (green) and PLPV-infected plants (red) harvested at 34 d.p.i. Bars depict standard deviations from three independent biological replicates and the statistical significance was tested using a paired *t*-test (* *p* < 0.05; ** *p* < 0.01).

As we observed significant alterations in the methylation state of the host rDNA promoter, we next checked whether infection by PLPV was linked to changes in the expression of genes involved in methylation/demethylation processes. Through an RT-qPCR approach, we compared, in PLPV-infected versus non-infected plants, the levels of transcripts derived from the following genes: MET1, CMT3, DRM2, RDR2, DCL3, AGO4, ROS1, DML2, DML3, and the core subunits of the plant-specific DNA-dependent RNA polymerases Pol IV and PolV, named NRPD1 and NRPE1, respectively. The results showed a significant increase the expression levels of demethylases ROS1, DML2, and DML3, whereas a decrease in the expression levels of MET1, the DNA methyltransferase responsible for the maintenance of CG methylation, was detected (Figure 2). Such disturbances in the balance of methylation/demethylation enzymes are remarkable and could be on their own sufficient to drive hypomethylation in the studied rDNA promoter region.

### 3.3. Hypomethylation of rDNA Promoter in PLPV-Infected Plants Is Linked to Increased Levels of rRNA Precursor Molecules

**Figure 3:**
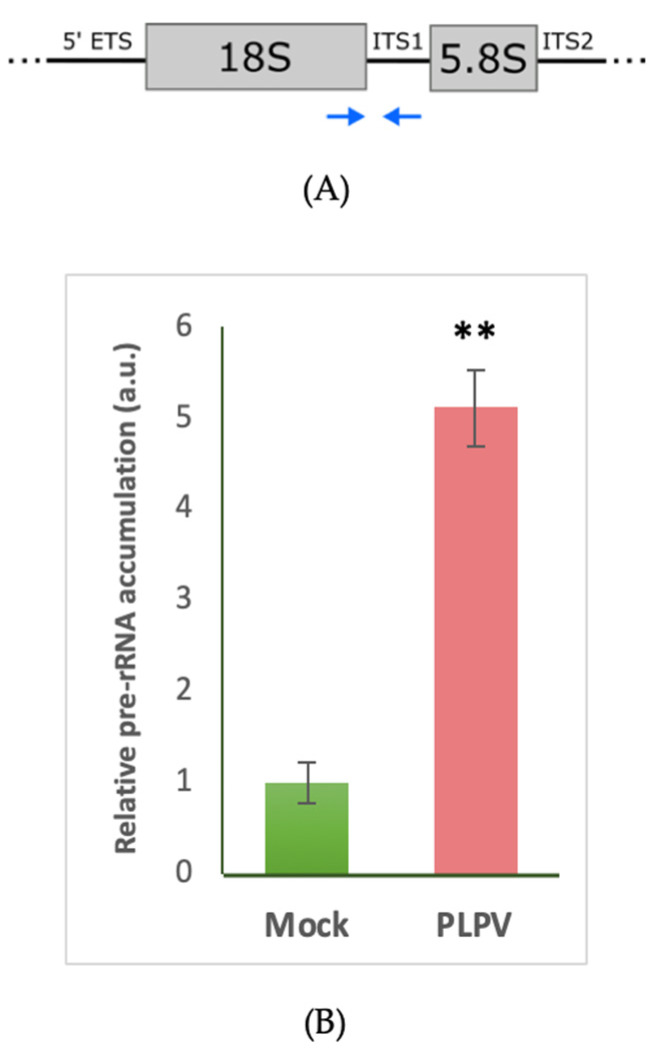
Relative accumulation of pre-rRNA. (**A**) Fragment of the rRNA unit. Blue arrows below depict the position of the primers used in the analysis. (**B**) RT-qPCR analysis of systemic leaves from mock-inoculated (green) and PLPV-infected (red) plants harvested at 34 d.p.i. Bars depict standard deviations from three independent biological replicates and the statistical significance was tested using a paired *t*-test (** *p* < 0.01).

In order to check whether the loss in methylated cytosines observed in the promoter region of rDNA in PLPV-infected versus non-infected plants had any effect on rRNA production, comparison of pre-rRNA levels was performed through RT-qPCR. Primers selected for this approach embraced a region of the 18S rRNA and the other of the ITS1 in such a way that a segment of the pre-rRNA would be amplified (Figure 3A). The measurements showed a remarkable increase in pre-rRNA levels in PLPV-infected tissue that exceeded by 5-fold those found in non-infected tissue (Figure 3B). These results showed a positive correlation between the hypomethylated state of rDNA promoter and pre-rRNA levels, suggesting that epigenetic marks in the analyzed promoter region have a functional outcome in rDNA transcription.

### 3.4. The PLPV VSR on Its Own Is Not Able to Incite Reduction in Cytosine Methylation

**Figure 4:**
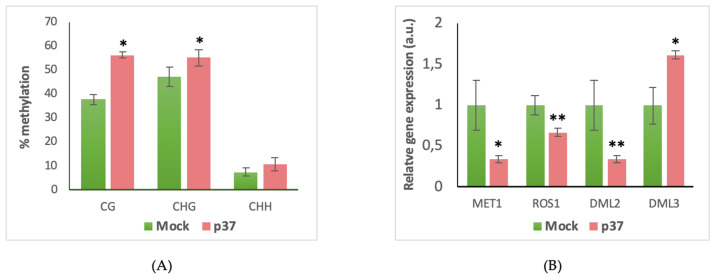
Analysis of the potential implication of the VSR p37 in rDNA promoter hypomethylation in *N. benthamiana* plants. (**A**) Differential DNA methylation levels between mock (green) and p37 (red) agroinfiltrated plants in the CG, CHG, and CHH sequence contexts. Error bars depict the standard deviations from two independent biological replicates. (**B**) RT-qPCR to estimate the relative expression levels of the genes involved in methylation/demethylation with imbalances in PLPV-infected plants. Data are representative of three biological replicates. The statistical significance was tested using a paired *t*-test (* *p* < 0.05; ** *p* < 0.01).

As virtually all plant viruses, PLPV produces a VSR, protein p37 [36]. As other VSRs encoded by RNA viruses, p37 is able to efficiently block post-transcriptional gene silencing (PTGS) [36]. However, some VSRs encoded by DNA viruses and at least one encoded by an RNA virus have been proposed to inhibit transcriptional gene silencing (TGS) by interfering with DNA methylation [26,50,51,52]. Giving these precedents, we wondered whether PLPV p37 itself could lessen DNA methylation levels. To approach this question, we first tried to generate transgenic *N. benthamiana* plants transformed with the PLPV p37 gene in order to compare the methylation status of the 45S rDNA with regard to control plants (transformed with an empty binary vector). However, no single plant transformed with the p37 gene could be recovered from distinct transformation attempts in contrast with the efficient production of plants transformed with an empty construct or with other types of constructs (Supplementary Figure S2). This observation suggested that the transgenic expression of the p37 gene provokes strong deleterious and insurmountable effects on plant regeneration and/or development. In view of this, we decided to transiently express p37 in *N. benthamiana* leaves via agroinfiltration. In parallel, *N. benthamiana* leaves were infiltrated with *A. tumefaciens* cultures transformed with an empty vector to be used as a mock control (Supplementary Figure S3). Leaf material was collected 14 d.p.if. since longer periods resulted in leaf senescence. Genomic DNA was extracted from the distinct samples (two biological replicates), subjected to bisulfite conversion, and used to PCR amplify the promoter region of the 45S rDNA transcriptional unit (positions −94 to +98, as in previous assays). Cloning and sequencing of a total of 27 clones revealed a significant increase in the level of cytosine methylation at the CG and CHG context between both types of samples (Figure 4A). Moreover, analysis of the expression levels of genes of the methylation/demethylation machinery exhibiting imbalances in PLPV-infected versus non-infected *N. benthamiana* (Figure 2) showed a disparate scenario: An increase and a decrease, respectively, of MET1 and DML3 mRNA levels were detected in p37-expressing leaves as found in PLPV-infected material, but, in contrast with the latter one, the mRNA levels of ROS1 and DML2 were significantly reduced (Figure 4B). These results suggested, on the one hand, that hypomethylation of rDNA in PLPV-infected plants depends, to a considerable extent, on the augmentation of ROS1 and/or DML2 levels and, on the other, that the VSR of PLPV is not the main factor responsible for the cytosine hypomethylation of host rDNA detected in PLPV-infected plants. 

## 4. Discussion

Different studies have shown changes in the epigenetic marks of plant genomes associated with infection by a variety of pathogens, particularly bacteria (*Pseudomonas syringae*), but also nematodes, fungi, and DNA viruses [53]. However, as indicated in the introduction section, much less is known on the effects of RNA viruses on the plant epigenome [30,31,32]. In this work, we analyzed whether PLPV infection may induce alterations in the methylation state of the 45S rDNA transcriptional unit and, more specifically, of its promoter. The choice of this target sequence was based on previous reports showing that this section of the genomic DNA may undergo significant changes in the extent of cytosine methylation under specific developmental or environmental signaling cues [40,54]. Such epigenetic modification is one of the mechanisms that contributes to the precise regulation of transcription levels of rRNA genes that are arranged in multiple tandem copies in nucleolar regions of the plant cell nucleus [55]. We showed that PLPV infections result in significant alterations on host rDNA methylation patterns, which involve hypomethylation at the CG sites of the promoter (Figure 1). This probably implies an intricate mechanism for regulation that may entail changes in the level/activity of both DNA methyltransferases and DNA glycosylases. Supporting this view, a reduction and an increase, respectively, in the expression levels of the maintenance methyltransferase MET1 and of demethylases ROS1, DML2, and DML3 were detected in PLPV-infected leaves in comparison with mock controls (Figure 2). These results suggest that the virus infection is able to manipulate key enzymes of the methylation/demethylation pathways most likely to influence host gene expression. In the same line, alterations in the levels of maintenance DNA methyltransferases and demethylases have been reported in plants infected by some viruses with DNA genomes, particularly geminiviruses [51], suggesting that distinct types of viral agents may take control of host gene expression through similar strategies. Moreover, as in the case of PLPV, a decrease in the number of cytosine residues that are methylated at CG sites, with no significant changes at non-CG sites, was detected in plants infected by such geminiviruses, which was consistent with a concomitant reduction in MET1 levels [51].

The introduction of methyl groups onto cytosines may result in the silencing of gene expression if they are in or close to promoter regions [21,22]. Conversely, hypomethylation frequently leads to gene activation. As indicated previously, the epigenetic on/off switch is one of the mechanisms that regulates the transcriptional activity of rRNA genes. Polymerase I recognizes the promoter of active rDNA genes to transcribe the pre-rRNAs from which mature rRNAs are gradually released after a complex series of processing steps [48,49]. Hypomethylation of the rDNA promoter in PLPV-infected samples correlated with an augmentation in pre-rRNA accumulation levels (Figure 3), which suggested that the virus infection contributes to the transcriptional activation of normally silenced rDNA units. The biological meaning of such an observation is uncertain though as virus multiplication relies on active translation of essential viral genes, it could be envisioned as part of the strategy of the virus to favor its multiplication [56]. Indeed, it has been reported that viral infections induce production of a class of endogenous siRNAs that may target rRNAs, which could constitute a defensive response of the host [57]. However, such siRNAs seem to lack silencing activity [57], most likely because maintenance or enhancement of rRNA levels may also promote translation of components involved in host defense. Thus, one of the possible outcomes of the interaction between the virus and the plant, i.e., variations in rRNA contents, may have both positive and negative effects on each of the parties involved in the arms race, further underlining the complexity of their relationship. An increment on pre-rRNA levels associated to diminished methylation of the rDNA promoter has also been reported in plants infected by a viroid (subviral infectious agents consisting of naked, non-coding, circular RNAs) [58]. In that case, alterations in the DNA methylation state of rDNA were suggested to be connected to viroid pathogenesis [58]. However, the asymptomatic condition of PLPV infections makes such a suggestion unlikely and rather points to a host response, which might be general, to viral/subviral entities.

VSRs counteract the antiviral RNA silencing response of the host that occurs at the post-transcriptional level [59]. This response is triggered by viral dsRNA molecules that are recognized by DCL enzymes to generate virus derived-siRNAs. One strand of these small duplexes is loaded into AGO-containing effector complexes and guides them towards cognate viral RNAs for degradation. Besides their role in blocking this process, some VSRs also seem able to modulate DNA methylation events. For instance, various VSRs encoded by geminiviruses have been shown to impair DNA methylation of the viral DNA genome, a host mechanism aimed at blocking viral replication, and, concomitantly, they also interfere with plant DNA methylation [26,50,51], thus acting as TGS suppressors. For PLPV, we could not confirm that p37, the PTGS suppressor of the virus, is the viral product responsible for the alteration in host rDNA methylation (Figure 4), despite its presence in the nucleolus [43], where the rDNA concentrates. Other viral products, the viral genome itself, or the infectious process as a whole may cause the changes observed in cytosine methylation at the rDNA level.

DNA demethylation has been proposed to restrict the propagation of different types of pathogens most likely by activating stress response genes that frequently are endowed with transposable elements in their promoters, which are, in general, the preferred targets of demethylases [21,60,61]. As proposed previously, DNA demethylation may facilitate the recruitment of PolII directly and/or through transactivators [62]. Interestingly, our present results (and other´s results; [58]) suggest that DNA demethylation may also promote recruitment of PolI. Though we did not investigate PolII-mediated transcription in the present work, studies of alterations in the plant epigenetic landscape associated to infections by a couple of plant RNA viruses, *Cucumber mosaic virus* (CMV, genus *Cucumovirus*, family *Bromoviridae*) and *Tobacco rattle virus* (TRV, genus *Tobravirus*, family *Virgaviridae*), have been recently performed [30,31]. The results have shown the presence of differentially methylated regions (DMRs) in the host genome that corresponded to both hypermethylated and hypomethylated segments. Though a certain correlation between hypomethylated promoters and higher expression levels of the corresponding genes was found in CMV-infected tissue, the genes following this pattern were not significantly involved in any particular cellular process or metabolic pathway and, thus, the biological meaning of the finding was unclear [31]. In the second case, TRV-responsive DMRs were also identified and though either hyper- or hypomethylation of cytosines was detected within such DRMs, hypomethylation was slightly more frequent at least in DMRs located upstream of the transcriptional start site of protein-coding genes [30]. Similar to what has been found in the present work and in plants infected by geminiviruses, a reduction in the expression levels of MET1 was detected in TRV-infected tissue, suggesting that this component of the methylation machinery might be a recurrent target of viral infections. In addition, it is worth mentioning that, in contrast to PLPV, CMV may be highly virulent and TRV generally causes mild symptoms, though both phenotypic effects and titers reached by these viruses depend on multiple factors, including the viral isolate, host species, or environmental conditions [63,64]. The establishment of potential correlations between viral accumulation and/or pathogenicity and the extent and nature of virus-induced epigenetic changes is an issue of unquestionable relevance and will require further investigations.

## 5. Conclusions

In summary, the results of this work have provided evidence for the occurrence of epigenetic changes in the plant rDNA promoter induced by PLPV infection. The hypomethylation at CG sites observed in this region correlates with an increase in pre-rRNA accumulation levels and seems to be connected with disturbances in the expression of methytransferases/demethylases. Such effects were not reproduced by the sole expression of the viral VSR, protein p37, indicating that other viral components or the viral infection as a whole might contribute to set patterns of DNA methylation in the invaded organism.

Finally, the impact of PLPV on epigenetic machinery reported here together with other recent reports [30,31] allows the inclusion of epigenetic pathways among the host biological processes altered by RNA viruses. Future research on this topic within the next few years is likely to substantially improve our understanding of how those changes influence the outcome of plant–virus interactions.

## Supplementary Materials

The following are available online at https://www.mdpi.com/2079-7737/9/5/91/s1, Table S1: List of primers used in this work, Figure S1: *Nicotiana benthamiana* plants infected with PLPV at 34 d.p.i., Figure S2: Phenotypic alterations in 35S::p37 transgenic plants, Figure S3: Analysis of p37 accumulation in *Nicotiana benthamiana* leaves agroinfiltrated with pMOG800-p37 or pMOG800, the empty binary vector (as control).

## References

[1] Hull R. (2014). Plant Virology.

[2] Lewsey M., Palukaitis P., Carr J.P. (2009). Plant–virus interactions: Defence and counter-defence. Annual Plant Reviews Volume 34: Molecular Aspects of Plant Disease Resistance.

[3] Wu X., Valli A., García J.A., Zhou X., Cheng X. (2019). The tug-of-war between plants and viruses: Great progress and many remaining questions. Viruses.

[4] Wang A. (2015). Dissecting the molecular network of virus-plant interactions: The complex roles of host factors. Annu. Rev. Phytopathol..

[5] Garcia-Ruiz H. (2019). Host factors against plant viruses. Mol. Plant Pathol..

[6] Garcia-Ruiz H. (2018). Susceptibility genes to plant viruses. Viruses.

[7] Han G.Z. (2019). Origin and evolution of the plant immune system. New Phytol..

[8] García J.A., Pallás V. (2015). Viral factors involved in plant pathogenesis. Curr. Opin. Virol..

[9] Whitham S.A., Yang C., Goodin M.M. (2006). Global impact: Elucidating plant responses to viral infection. Mol. Plant Microbe Interact..

[10] Oktem H., Eyidogan F., Selçuk F., Oz T., Teixeira da Silva J., Yücel M. (2008). Revealing response of plants to biotic and abiotic stresses with microarray technology. Genes Genomes Genom..

[11] Du J., Johnson L.M., Jacobsen S.E., Patel D.J. (2015). DNA methylation pathways and their crosstalk with histone methylation. Nat. Rev. Mol. Cell Biol..

[12] Eichten S.R., Schmitz R.J., Springer N.M. (2014). Epigenetics: Beyond chromatin modifications and complex genetic regulation. Plant Physiol..

[13] Feng S., Jacobsen S.E., Reik W. (2010). Epigenetic reprogramming in plant and animal development. Science.

[14] Matzke M.A., Kanno T., Matzke A.J.M. (2015). RNA-directed DNA methylation: The evolution of a complex epigenetic pathway in flowering plants. Annu. Rev. Plant Biol..

[15] Zhang H., Lang Z., Zhu J.-K. (2018). Dynamics and function of DNA methylation in plants. Nat. Rev. Mol. Cell Biol..

[16] Movahedi A., Sun W., Zhang J., Wu X., Mousavi M., Mohammadi K., Yin T., Zhuge Q. (2015). RNA-directed DNA methylation in plants. Plant Cell Rep..

[17] Matzke M.A., Mosher R.A. (2014). RNA-directed DNA methylation: An epigenetic pathway of increasing complexity. Nat. Rev. Genet..

[18] Gong Z., Morales-Ruiz T., Ariza R.R., Roldán-Arjona T., David L., Zhu J.K. (2002). ROS1, a repressor of transcriptional gene silencing in *Arabidopsis*, encodes a DNA glycosylase/lyase. Cell.

[19] Penterman J., Zilberman D., Huh J.H., Ballinger T., Henikoff S., Fischer R.L. (2007). DNA demethylation in the *Arabidopsis* genome. Proc. Natl. Acad. Sci. USA.

[20] Zhu J.K. (2009). Active DNA demethylation mediated by DNA glycosylases. Annu. Rev. Genet..

[21] Baulcombe D.C., Dean C. (2014). Epigenetic regulation in plant responses to the environment. Cold Spring Harb. Perspect. Biol..

[22] Dowen R.H., Pelizzola M., Schmitz R.J., Lister R., Dowen J.M., Nery J.R., Dixon J.E., Ecker J.R. (2012). Widespread dynamic DNA methylation in response to biotic stress. Proc. Natl. Acad. Sci. USA.

[23] Ding B., Wang G.-L. (2015). Chromatin versus pathogens: The function of epigenetics in plant immunity. Front. Plant Sci..

[24] Butterbach P., Verlaan M.G., Dullemans A., Lohuis D., Visser R.G.F., Bai Y., Kormelink R. (2014). *Tomato yellow leaf curl virus* resistance by Ty-1 involves increased cytosine methylation of viral genomes and is compromised by *Cucumber mosaic virus* infection. Proc. Natl. Acad. Sci. USA.

[25] Raja P., Sanville B.C., Buchmann R.C., Bisaro D.M. (2008). Viral genome methylation as an epigenetic defense against geminiviruses. J. Virol..

[26] Yang L.-P., Fang Y.-Y., An C.-P., Dong L., Zhang Z.-H., Chen H., Xie Q., Guo H.-S. (2013). C2-mediated decrease in DNA methylation, accumulation of siRNAs, and increase in expression for genes involved in defense pathways in plants infected with *Beet severe curly top virus*. Plant J. Cell Mol. Biol..

[27] Kanazawa A., Inaba J., Shimura H., Otagaki S., Tsukahara S., Matsuzawa A., Kim B.M., Goto K., Masuta C. (2011). Virus-mediated efficient induction of epigenetic modifications of endogenous genes with phenotypic changes in plants. Plant J..

[28] Kon T., Yoshikawa N. (2014). Induction and maintenance of DNA methylation in plant promoter sequences by *Apple latent spherical virus*-induced transcriptional gene silencing. Front. Microbiol..

[29] Otagaki S., Kawai M., Masuta C., Kanazawa A. (2011). Size and positional effects of promoter RNA segments on virus-induced RNA-directed DNA methylation and transcriptional gene silencing. Epigenetics.

[30] Diezma-Navas L., Pérez-González A., Artaza H., Alonso L., Caro E., Llave C., Ruiz-Ferrer V. (2019). Crosstalk between epigenetic silencing and infection by *Tobacco rattle virus* in *Arabidopsis*. Mol. Plant Pathol..

[31] Wang C., Wang C., Xu W., Zou J., Qiu Y., Kong J., Yang Y., Zhang B., Zhu S. (2018). Epigenetic changes in the regulation of *Nicotiana tabacum* response to *Cucumber mosaic virus* infection and symptom recovery through single-base resolution methylomes. Viruses.

[32] Wang C., Wang C., Zou J., Yang Y., Li Z., Zhu S. (2019). Epigenetics in the plant–virus interaction. Plant Cell Rep..

[33] Scheets K., Jordan R., White K.A., Hernández C. (2015). *Pelarspovirus*, a proposed new genus in the family *Tombusviridae*. Arch. Virol..

[34] Castaño A., Hernández C. (2005). Complete nucleotide sequence and genome organization of *Pelargonium line pattern virus* and its relationship with the family *Tombusviridae*. Arch. Virol..

[35] Castaño A., Ruiz L., Hernández C. (2009). Insights into the translational regulation of biologically active open reading frames of *Pelargonium line pattern virus*. Virology.

[36] Pérez-Cañamás M., Hernández C. (2015). Key importance of small RNA binding for the activity of a glycine-tryptophan (GW) motif-containing viral suppressor of RNA silencing. J. Biol. Chem..

[37] Alonso M., Borja M. (2005). High incidence of *Pelargonium line pattern virus* infecting asymptomatic *Pelargonium* spp. in Spain. Eur. J. Plant Pathol..

[38] Ivars P., Alonso M., Borja M., Hernández C. (2004). Development of a non-radioactive dot-blot hybridization assay for the detection of *Pelargonium flower break virus* and *Pelargonium line pattern virus*. Eur. J. Plant Pathol..

[39] Pérez-Cañamás M., Blanco-Pérez M., Forment J., Hernández C. (2017). *Nicotiana benthamiana* plants asymptomatically infected by *Pelargonium line pattern virus* show unusually high accumulation of viral small RNAs that is neither associated with DCL induction nor RDR6 activity. Virology.

[40] Tucker S., Vitins A., Pikaard C.S. (2010). Nucleolar dominance and ribosomal RNA gene silencing. Curr. Opin. Cell Biol..

[41] Castaño A., Hernández C. (2007). Biological activity of transcripts from cDNA of *Pelargonium line pattern virus*. Acta Virol..

[42] Blanco-Pérez M., Hernández C. (2016). Evidence supporting a premature termination mechanism for subgenomic RNA transcription in *Pelargonium line pattern virus*: Identification of a critical long-range RNA-RNA interaction and functional variants through mutagenesis. J. Gen. Virol..

[43] Pérez-Cañamás M., Hernández C. (2018). New insights into the nucleolar localization of a plant RNA virus-encoded protein that acts in both RNA packaging and RNA silencing suppression: Involvement of importins alpha and relevance for viral infection. Mol. Plant Microbe Interact..

[44] Li L.C., Dahiya R. (2002). MethPrimer: Designing primers for methylation PCRs. Bioinforma. Oxf. Engl..

[45] Hetzl J., Foerster A.M., Raidl G., Mittelsten Scheid O. (2007). CyMATE: A new tool for methylation analysis of plant genomic DNA after bisulphite sequencing. Plant J. Cell Mol. Biol..

[46] Verwoerd T.C., Dekker B.M.M., Hoekema A. (1989). A small-scale procedure for the rapid isolation of plant RNAs. Nucleic Acids Res..

[47] Liu D., Shi L., Han C., Yu J., Li D., Zhang Y. (2012). Validation of reference genes for gene expression studies in virus-infected *Nicotiana benthamiana* using quantitative Real-Time PCR. PLoS ONE.

[48] McStay B., Grummt I. (2008). The epigenetics of rRNA genes: From molecular to chromosome biology. Annu. Rev. Cell Dev. Biol..

[49] Pikaard C.S. (2000). The epigenetics of nucleolar dominance. Trends Genet..

[50] Buchmann R.C., Asad S., Wolf J.N., Mohannath G., Bisaro D.M. (2009). Geminivirus AL2 and L2 proteins suppress transcriptional gene silencing and cause genome-wide reductions in cytosine methylation. J. Virol..

[51] Rodríguez-Negrete E., Lozano-Durán R., Piedra-Aguilera A., Cruzado L., Bejarano E.R., Castillo A.G. (2013). Geminivirus Rep protein interferes with the plant DNA methylation machinery and suppresses transcriptional gene silencing. New Phytol..

[52] Yang L., Xu Y., Liu Y., Meng D., Jin T., Zhou X. (2016). HC-Pro viral suppressor from *Tobacco vein banding mosaic virus* interferes with DNA methylation and activates the salicylic acid pathway. Virology.

[53] Alonso C., Ramos-Cruz D., Becker C. (2019). The role of plant epigenetics in biotic interactions. New Phytol..

[54] Östlund Farrants A.-K., Göndör A. (2017). Epigenetic regulation of nucleolar functions. Chromatin Regulation and Dynamics.

[55] Sáez-Vásquez J., Delseny M. (2019). Ribosome biogenesis in plants: From functional 45S ribosomal DNA organization to ribosome assembly factors. Plant Cell.

[56] Jan E., Mohr I., Walsh D. (2016). A cap-to-tail guide to mRNA translation strategies in virus-infected cells. Annu. Rev. Virol..

[57] Cao M., Du P., Wang X., Yu Y.-Q., Qiu Y.H., Li W., Gal-On A., Zhou C., Li Y., Ding S.W. (2014). Virus infection triggers widespread silencing of host genes by a distinct class of endogenous siRNAs in *Arabidopsis*. Proc. Natl. Acad. Sci. USA.

[58] Martinez G., Castellano M., Tortosa M., Pallas V., Gomez G. (2014). A pathogenic non-coding RNA induces changes in dynamic DNA methylation of ribosomal RNA genes in host plants. Nucleic Acids Res..

[59] Csorba T., Kontra L., Burgyán J. (2015). Viral silencing suppressors: Tools forged to fine-tune host-pathogen coexistence. Virology.

[60] Deleris A., Halter T., Navarro L. (2016). DNA methylation and demethylation in plant immunity. Annu. Rev. Phytopathol..

[61] Le T.N., Schumann U., Smith N.A., Tiwari S., Au P.C.K., Zhu Q.H., Taylor J.M., Kazan K., Llewellyn D.J., Zhang R. (2014). DNA demethylases target promoter transposable elements to positively regulate stress responsive genes in *Arabidopsis*. Genome Biol..

[62] Yu A., Lepère G., Jay F., Wang J., Bapaume L., Wang Y., Abraham A.L., Penterman J., Fischer R.L., Voinnet O. (2013). Dynamics and biological relevance of DNA demethylation in *Arabidopsis* antibacterial defense. Proc. Natl. Acad. Sci. USA.

[63] Palukaitis P., García-Arenal F. (2003). *Cucumoviruses*. Adv. Virus Res..

[64] Ratcliff F., Martin-Hernandez A.M., Baulcombe D.C. (2001). Technical advance: *Tobacco rattle virus* as a vector for analysis of gene function by silencing. Plant J..

