# Supplementary material for: Epigenetic Changes in Host Ribosomal DNA Promoter Induced by an Asymptomatic Plant Virus Infection
Source: Biology (Basel). 2020 Apr 28;9(5):91. doi: 10.3390/biology9050091 (PMC7285159; doi:10.3390/biology9050091)
Supplement: Supplementary file 1 [file biology-09-00091-s001.pdf]

Supplementary Table S1. List of primers used in this work:

Gene	Position	Genbank Acc. no.[1]	Strand	Sequence
**MET1**	4498-4598	*	S	5′ GACAGGATTGTTACAGTTCGTGAATG 3′
			AS	5′ TTCCTATTTGCCTGTGCTTGTG 3′
**CMT3b**	2056-2184	KC453971.1	S	5′ TCGGTGGAGAATAATGAGCAAAGAG 3′
			AS	5′ CAGAGGACGATGATCATAGAGGACAC 3′
**DRM2**	526-625	*	S	5′ GGATACCCCATGGAGGAGGTT 3′
			AS	5′ TTGCCATTTGAGCAGCACAT 3′
**RDR2**	2456-2555	AY722009.1	S	5′ ATGAATGCTCCGGTGGTGAT 3′
			AS	5′ CCAGTGTAGTCCATGGGTGTCA 3′
**DCL3**	4770-4949	*	S	5′ ACTTGTTGAATGCGGTGAAG 3′
			AS	5′ CCCCTGTCGTTCTAGCTCAT 3′
**AGO4a**	2555-2654	DQ321490.1	S	5′ TATCCATTGTGGCTCCGGTAA 3′
			AS	5′ CCATGGCTTGACGATGTCTCT 3′
**ROS1**	4457-4555	AB778815.1	S	5′ TGCCCTTACCTCAGGCTGAT 3′
			AS	5′ TCTACGATGGGCTCTGGTGTT 3′
**DML2**	5185-5284	*	S	5′ CCACGGCGGACTGTCTACTT 3′
			AS	5′ CACAAACAAACCCTCTCCAAAAG 3′
**DML3**	4002-4101	*	S	5′ TCTTACCGAAAGAAATAGGGATTCTG 3′
			AS	5′ AACATTACCCTGTCCTCGACCTT3′
**Pol IV**	2216-2315	*	S	5′ CATTTAGAATGCCGCGTCAAC 3′
			AS	5′ GAACCAGGACGTTCAGGAACA 3′
**Pol V**	5497-5596	*	S	5′ ATGCATCAGACAGGGTACAATGA 3′
			AS	5′ TGACAGCTTTATCGGGATGGT 3′
**Pre-rRNA**	3245-3344	KP824745.1	S	5′ ACCTGCGGAAGGATCATTGT 3′
			AS	5′ GACGGAGGCCGAAGCAT 3′
**PP2A**	996-1095	MF996339.1	S	5′ ACTTGGTGCCCTTTGTATGC 3′
			AS	5′ TGGACCAAATTCTTCTGCAA 3′

[1] Genbank accession number of the genes used in the analysis. The sequence of the genes marked with an asterisk was obtained from *Nicotiana benthamiana* genome database (https://benthgenome.qut.edu.au).

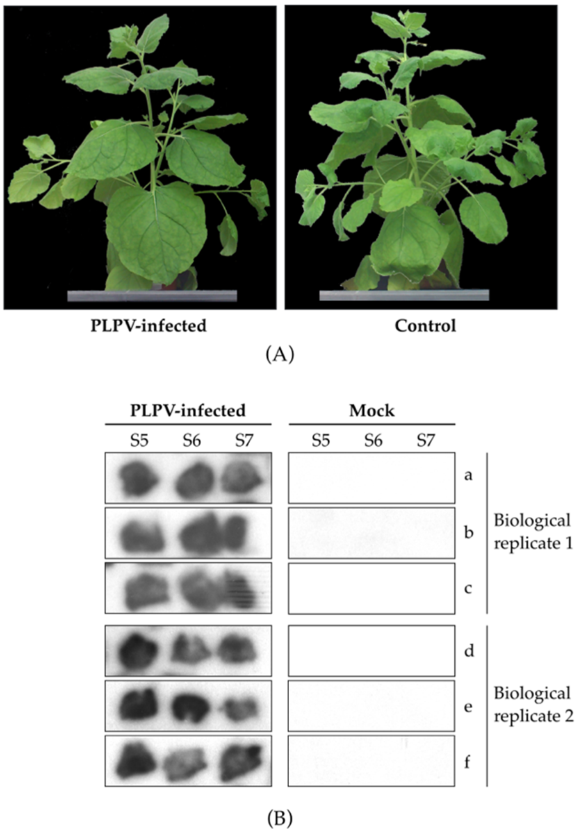

Supplementary Figure S1. *Nicotiana benthamiana* plants infected with PLPV at 34 d.p.i. (A) Overall phenotype of PLPV-infected and non-infected (control) plants. Virus-infected plants do not show any morphological or development alteration. (B) Tissue printing hybridization of systemic leaves 5, 6 and 7 from PLPV- and mock-inoculated plants using a PLPV-specific ^{32}P-labeled riboprobe. Systemic leaves from three independent plants were used for each biological replicate.

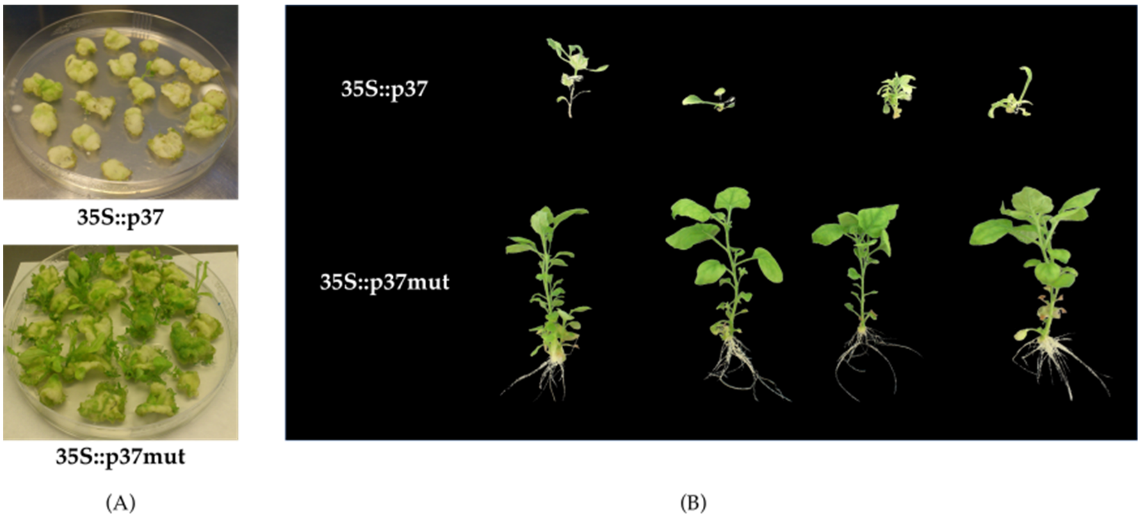

Supplementary Figure S2. Phenotypic alterations in 35S::p37 transgenic plants. *Nicotiana benthamiana* transgenic plants expressing p37, the VSR of PLPV, were generated from callus tissues by *Agrobacterium*-mediated transformation. Plants transformed with a p37 mutant (p37mut) without silencing suppressor activity (p37-W_{28}; [34]) were used as a control. (A) Embryogenic callus on the selection medium (50 mg/l kanamycin). (B) Transgenic plants expressing p37 or the mutant version of the protein. Constitutive expression of p37 produced deleterious effects, such as the absence of roots, leaf morphology alterations or chlorosis, so 35S::p37 transgenic plants could not be regenerated.

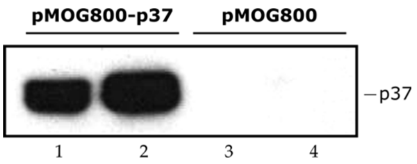

Supplementary Figure S3. Analysis of p37 accumulation in *Nicotiana benthamiana* leaves agroinfiltrated with pMOG800-p37 or pMOG800, the empty binary vector (as control). Leaves from six independent plants were pooled and protein extracts from two biological replicates were analysed by Western blot using an anti-p37 antibody.
